# Supplementary material for: Noninvasive liquid diet delivery of stable isotopes into mouse models for deep metabolic network tracing
Source: Nat Commun. 2017 Nov 21;8:1646. doi: 10.1038/s41467-017-01518-z (PMC5696342; doi:10.1038/s41467-017-01518-z)
Supplement: Supplementary file 1 — Supplementary Information [file 41467_2017_1518_MOESM1_ESM.pdf]

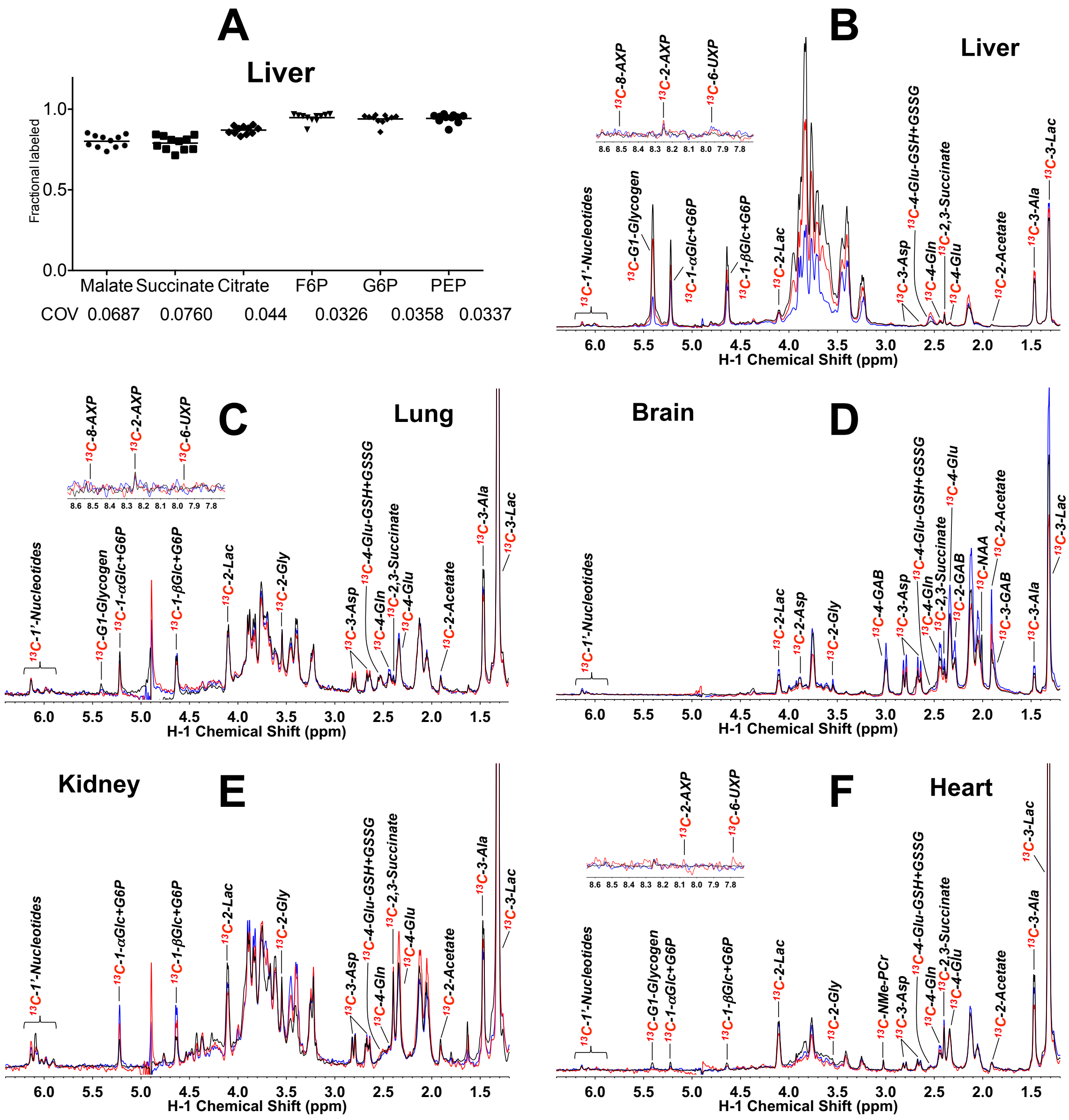

Supplementary Figure 1: NMR analysis demonstrates reproducibility of $^{13}C_6$-glucose incorporation into metabolites in 5 mouse organs using the liquid diet approach.

(**A**) Dot plot of fractional labeled glycolysis and Krebs cycle metabolites from 10 mouse livers and their corresponding coefficient of variance (CoV). **B-F** are respectively 1D $^{1}H\{^{13}C\}$ HSQC NMR spectra of polar extracts of liver (**B**), lung(**C**), brain (**D**), kidney (**E**), and heart (**F**) from three different wild type NSG mice fed on the $^{13}C_6$-glucose enriched liquid diet. The insets in **B**, **C**, and **F** display expanded spectral regions of nucleobases. These spectra were normalized to protein weight and overlaid to demonstrate good reproducibility of ^{13}C labeling in metabolites. NMR data were recorded at 14.1 T, 15 °C on an Agilent DD2 spectrometer with a 3 mm inverse triple resonance cryoprobe, an acquisition time of 0.2 s, a 2 s recycle time, and adiabatic proton decoupling.

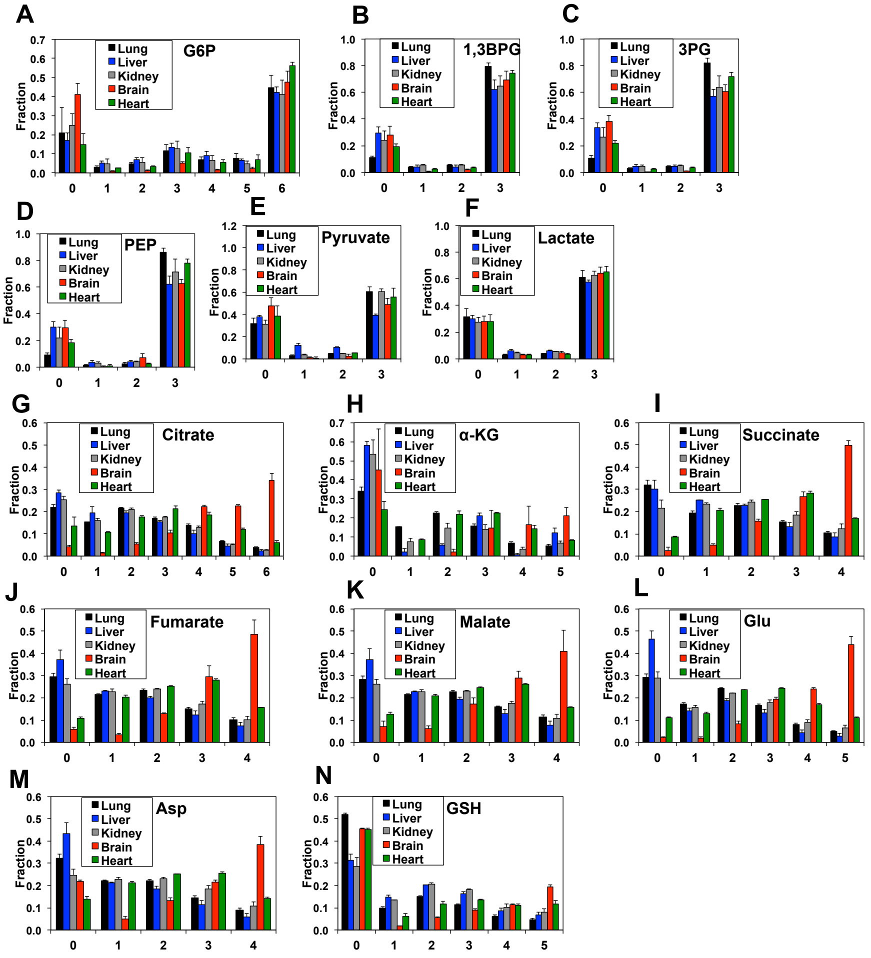

Supplementary Figure 2: IC-UHR-FTMS analysis indicates differential $^{13}C_6$-glucose metabolism in 5 mouse organs via glycolysis and the Krebs cycle.

The same polar extracts as in **Supplementary Figure 1** were analyzed by ion chromatography on a Dionex IonPac AS11-HC-4 µm RFIC&HPIC (2×250 mm) column (Thermo Scientific) coupled to a Thermo Orbitrap Fusion Tribrid mass spectrometer at a resolution of 450,000 at *m/z* of 200. Metabolites were identified with reference to standards, and their isotopologues quantified using Thermo TraceFinder (version 3.3) and subsequently corrected for natural abundance [1]. Shown in **A-F** and **G-N** are metabolites and their isotopologues that were derived from glycolysis and the Krebs cycle, respectively. G6P: glucose-6-phosphate; 1,3BPG: 1,3-bisphophoglycerate; 3PG: 3-phosphoglycerate; PEP: phosphoenolpyruvate; α-KG: α-ketoglutarate; GSH: reduced glutathione. The x-axis represents the number of ^{13}C atoms (i) present in each compound, represented in the text as $^{13}C_i$. Values shown were mean ± SEM (n=3).

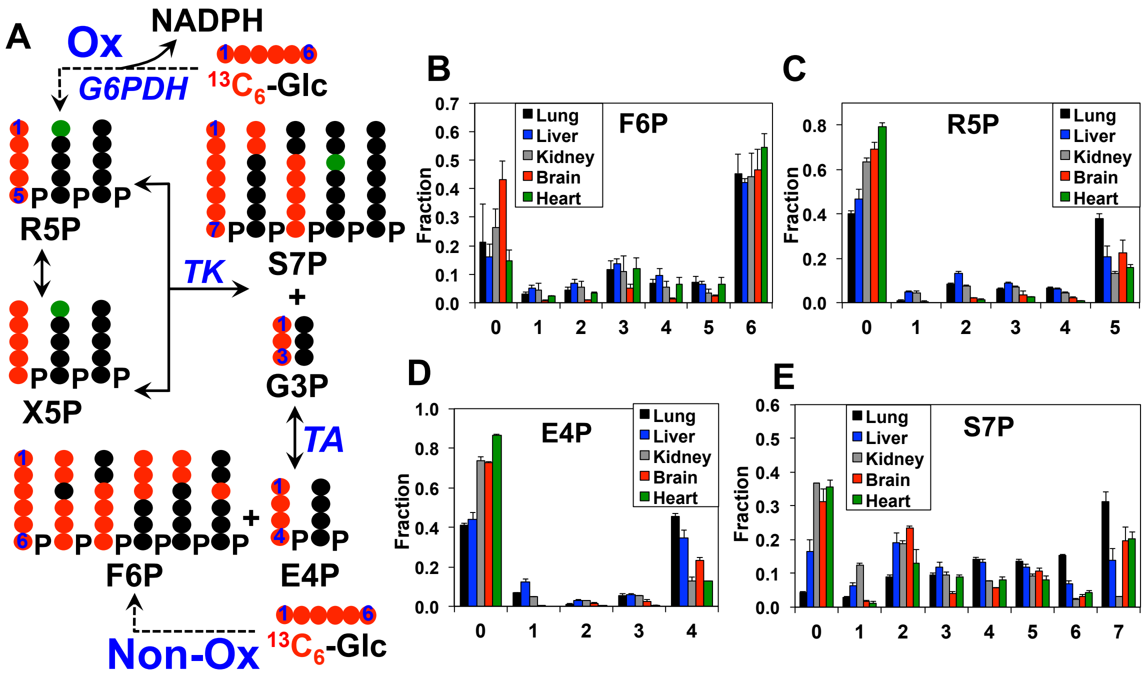

Supplementary Figure 3: IC-UHR-FTMS analysis indicates differential $^{13}C_6$-glucose metabolism in 5 mouse organs via the pentose phosphate pathway. The polar metabolites were analyzed by IC-UHR-FTMS as described in **Supplementary Figure 2**. In the 5 organs, total ^{13}C enrichment in R5P, E4P, and S7P was higher (or lower as the unlabeled fraction **0**) in lung and liver than the other 3 organs (**C-E**; **F** false discovery q values for comparison of the **0** fraction for lung or liver versus other organs). In addition to the fully ^{13}C labeled species, the ^{13}C scrambled species of the PPP intermediates fructose-6-phosphate (F6P, **B**), ribose-5-phosphate (R5P, **C**), erythrose-4-phosphate (E4P, **D**), and sedoheptulose-7-phosphate (S7P, **E**) were observed. The extent of ^{13}C scrambling in S7P (**1-6**, **E**) was most extensive and variable among organs (from a total of 44.1±4.2% for heart to 69.6±1.7% for liver), reflecting high and variable activity of the forward and/or reverse non-oxidative branch of PPP (**Non-Ox**) catalyzed by transketolase (TK) and transaldolase (TA). ●: ^{12}C; ●: ^{13}C from oxidative branch (**Ox**) + forward **Non-Ox** PPP; ●: ^{13}C from reverse **Non-Ox** PPP; E4P: erythrose-4-phosphate; X5P: xylulose-5-phosphate; G6PDH: glucose-6-phosphate dehydrogenase. Not all possible ^{13}C-isotopomers are shown. The x-axis shows the number of ^{13}C atoms present in each isotopologue represented in the text as $^{13}C_i$. Values shown were mean ± SEM (n=3).

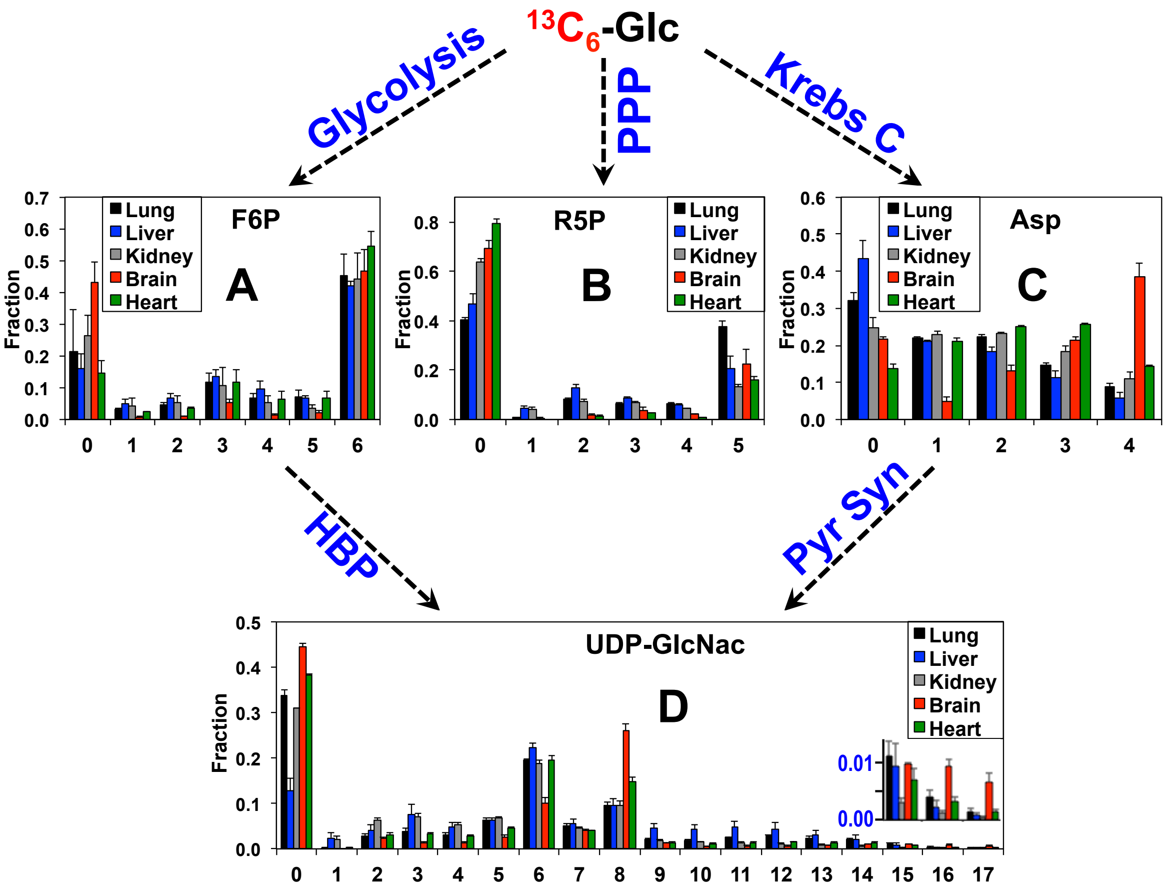

Supplementary Figure 4: IC-UHR-FTMS analysis indicates differential $^{13}C_6$-glucose metabolism in 5 mouse organs via the pentose phosphate pathway. The polar metabolites were analyzed by IC-UHR-FTMS as described in **Supplementary Figure 2**. In the 5 organs, total ^{13}C enrichment in R5P, E4P, and S7P was higher (or lower as the unlabeled fraction **0**) in lung and liver than the other 3 organs (**C-E**; **F** false discovery q values for comparison of the **0** fraction for lung or liver versus other organs). In addition to the fully ^{13}C labeled species, the ^{13}C scrambled species of the PPP intermediates fructose-6-phosphate (F6P, **B**), ribose-5-phosphate (R5P, **C**), erythrose-4-phosphate (E4P, **D**), and sedoheptulose-7-phosphate (S7P, **E**) were observed. The extent of ^{13}C scrambling in S7P (**1-6**, **E**) was most extensive and variable among organs (from a total of 44.1±4.2% for heart to 69.6±1.7% for liver), reflecting high and variable activity of the forward and/or reverse non-oxidative branch of PPP (**Non-Ox**) catalyzed by transketolase (TK) and transaldolase (TA). ●: ^{12}C; ●: ^{13}C from oxidative branch (**Ox**) + forward **Non-Ox** PPP; ●: ^{13}C from reverse **Non-Ox** PPP; E4P: erythrose-4-phosphate; X5P: xylulose-5-phosphate; G6PDH: glucose-6-phosphate dehydrogenase. Not all possible ^{13}C-isotopomers are shown. The x-axis shows the number of ^{13}C atoms present in each isotopologue represented in the text as $^{13}C_i$. Values shown are mean ± SEM (n=3).

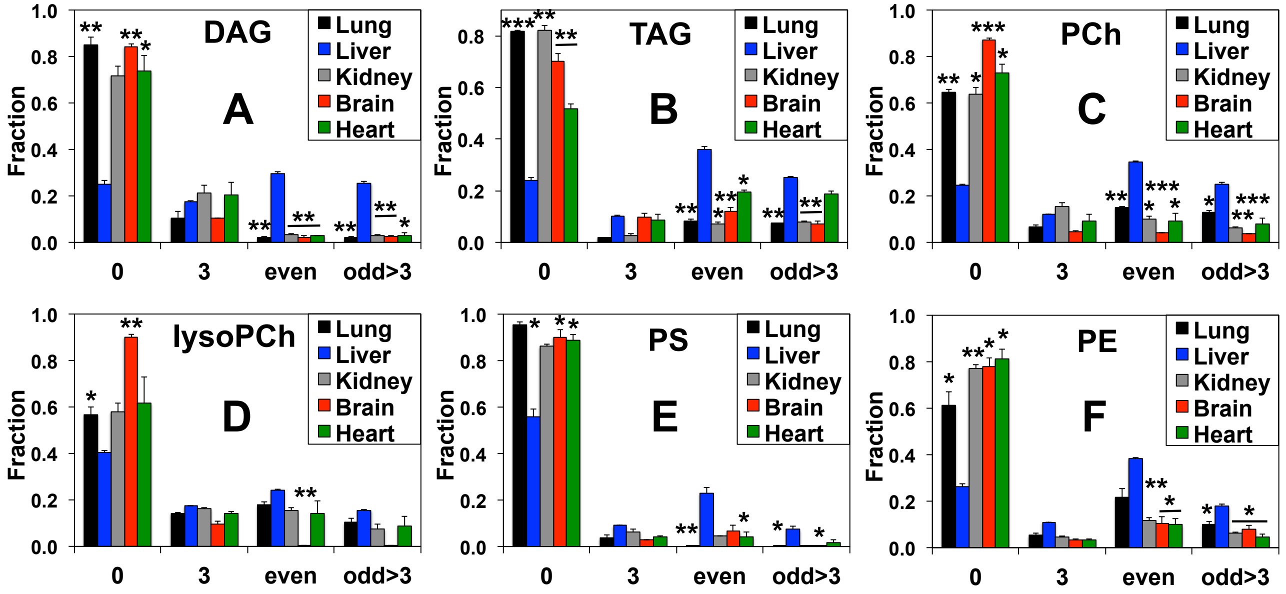

Supplementary Figure 5: UHR-FTMS analysis indicates differential $^{13}C_6$-glucose metabolism in 5 mouse organs via de novo lipid synthesis pathways.

Tissue lipids extracts from the three liquid diet-labeled mice of **Supplementary Fig. 2** were analyzed by direct infusion nanoelectrospray (nESI) via an Advion Nanomate interfaced to a Thermo Orbitrap Fusion mass spectrometer. Lipids and their ^{13}C isotopologues were identified by accurate mass using PREMISE. Shown here are the ^{13}C isotopologue distributions of diacylglycerides (DAG, **A**), triacylglycerides (TAG, **B**), phosphatidylcholines (PCh, **C**), lyosphosphatidylcholines (LysoPCh, **D**), phosphatidylserines (PS, **E**), and phosphatidylethanolamines (PE, **F**). The varying extent of ^{13}C incorporation into these lipids illustrated the differential capacity for de novo lipid synthesis. 3: $^{13}C_3$-glycerol backbone; even: $^{13}C_{\text{even number}}$-fatty acyl chains; odd>3: $^{13}C_3$-glycerol backbone + $^{13}C_{\text{even number}}$-fatty acyl chains [2]. *, **, and *** denote false discovery q values for liver versus lung, kidney, brain, and heart; <0.05-0.01, <0.01-0.001, and <0.001, respectively. See Methods for details.

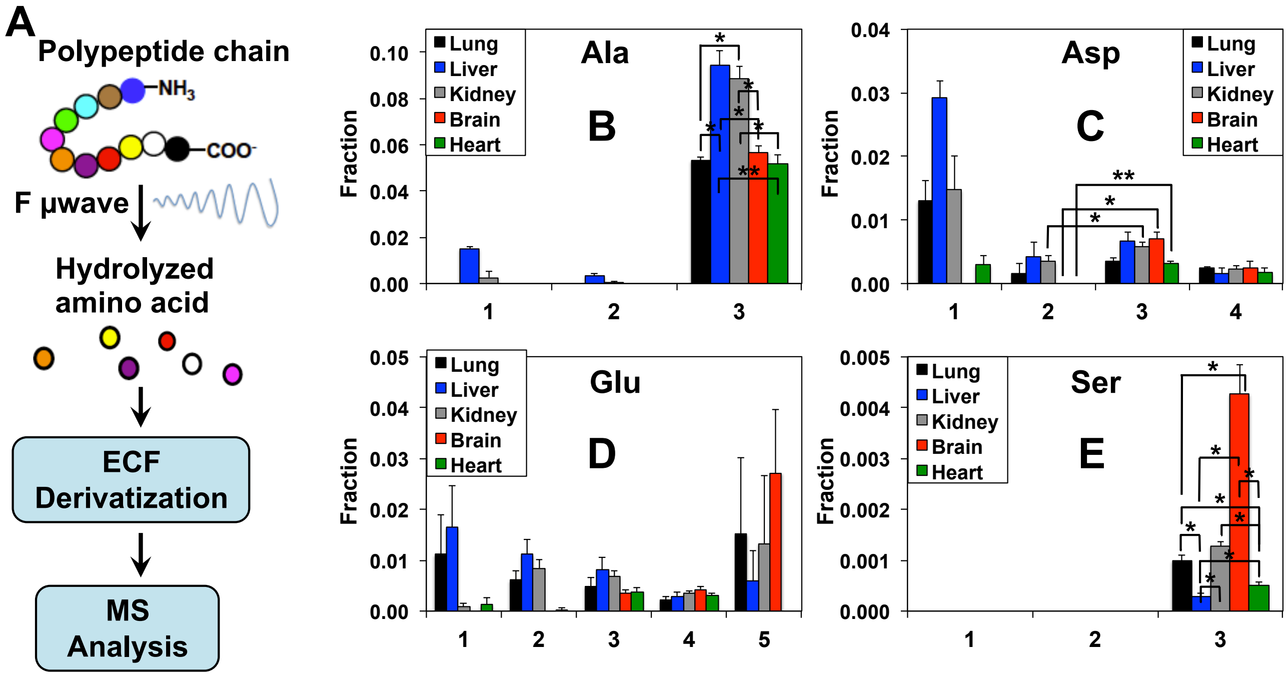

Supplementary Figure 6: ECF-UHR-FTMS analysis indicates differential $^{13}C_6$-glucose incorporation into proteins in 5 mouse organs. Extracted tissue proteins were hydrolyzed in 6N HCl by focused-beam microwave digestion (**µwave, F**). The free amino acids liberated were derivatized with ethyl chloroformate (ECF) before analysis by direct infusion FTMS [3] as in **Fig. S5**. **A** Schematic workflow for analyzing ^{13}C labeled amino acid incorporation into proteins. **B** to **E** Fractional enrichment distribution of ^{13}C isotopologues of the proteinaceous amino acids Ala, Asp, Glu, and Ser, respectively. The amino acids liberated were derivatized with ethyl chloroformate (ECF) before analysis by direct infusion FTMS [3] as in **Supplementary Figure 5**. The data indicate synthesis of these amino acids from $^{13}C_6$-glucose and subsequent incorporation into proteins in all 5 organs, with liver and kidney showing the highest extent of incorporation into Ala. Up to 10% of total Ala was ^{13}C labeled in the liver and kidney during the 24-h feeding period, reflecting a relatively high protein turnover rate in these two organs. The x-axis represents the number of ^{13}C atoms present in each isotopologue represented in the text as $^{13}C_i$. Values shown are mean ± SEM (n=3). * and ** in **B**, **E** denote false discovery q values for liver or kidney versus lung, brain, and heart; 0.01<q<0.05 and 0.001<q<<0.01, respectively. * in **C** denotes p values (<0.05-0.01) for $^{13}C_3$-(**3**) versus $^{13}C_2$-Asp (**2**) for kidney, brain, and heart.

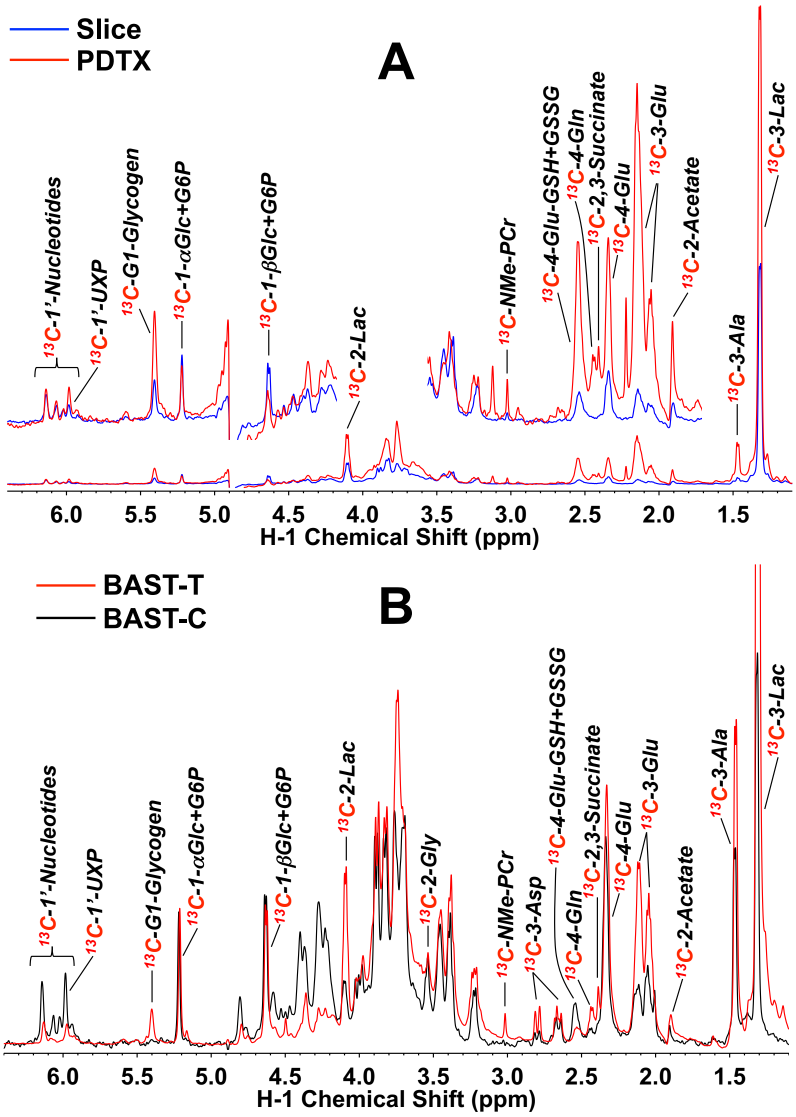

Supplementary Figure 7: NMR analysis demonstrates metabolic differences between *in vivo*, *ex vivo* and *in vitro* models of lung cancer.

1D $^{1}H\{^{13}C\}$ HSQC NMR spectra were recorded for the polar extracts of (**A**) PDTX of UK025 fed with $^{13}C_6$-Glc enriched liquid diet for 18 h and the *ex vivo* cancer tissue slices of the same patient cultured in $^{13}C_6$-Glc enriched DMEM medium for 24 h; (**B**) arsenite-transformed BEAS-2B (BAsT) cells cultured in $^{13}C_6$-Glc enriched DMEM medium for 24 h and the corresponding mouse xenograft fed with $^{13}C_6$-Glc enriched liquid diet for 18 h. The spectral intensities were normalized to protein weight for direct comparison.

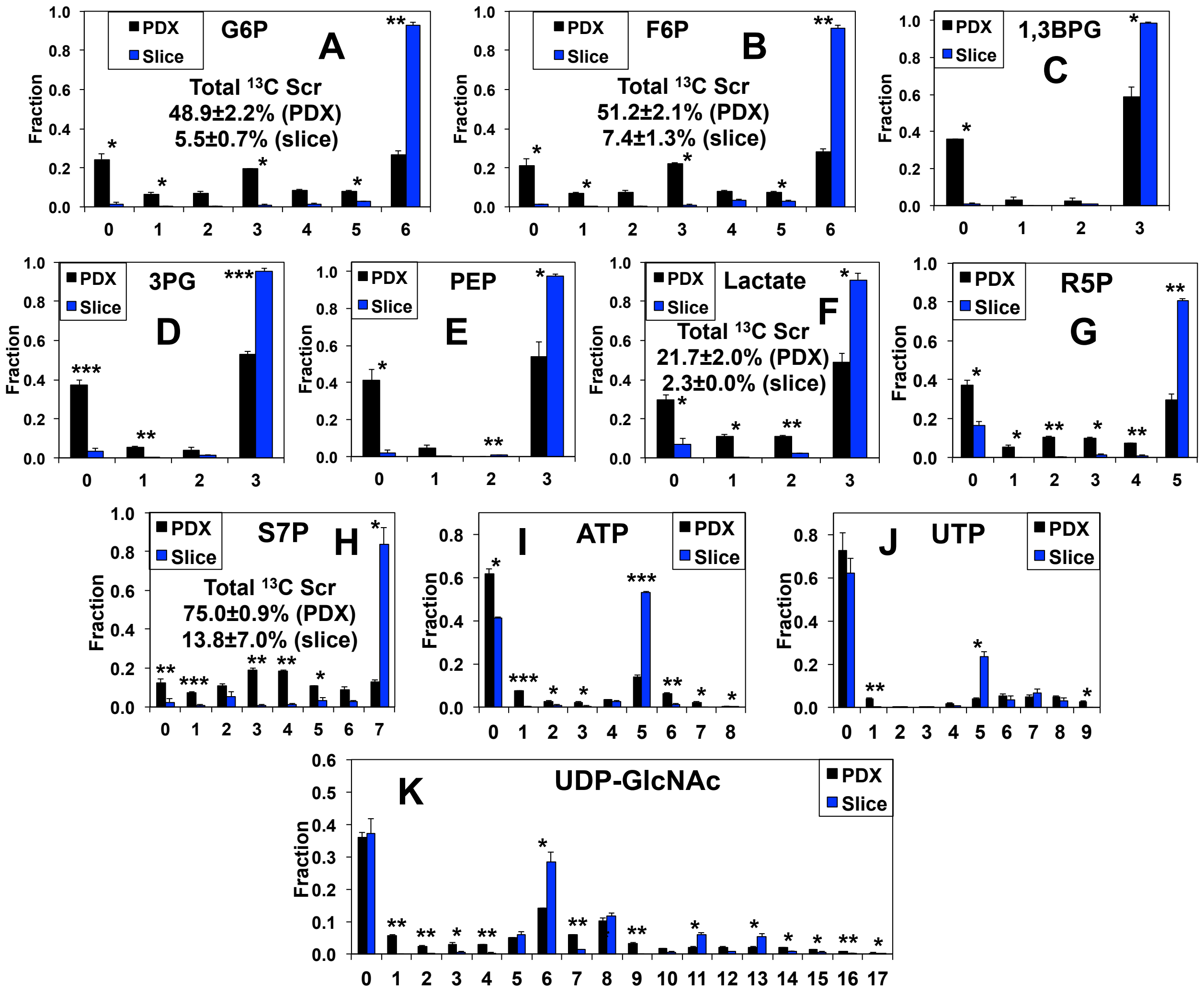

Supplementary Figure 8: IC-UHR-FTMS analysis showed differential extent of $^{13}C_6$-glucose incorporation into glycolytic metabolites, PPP metabolites, nucleotides, and sugar nucleotides in PDTX versus *ex vivo tissue slice* models of NSCLC. The polar metabolites from **Supplementary Fig. 7** were analyzed by IC-UHR-FTMS as described in **Supplementary Figure 2**. **A-F** showed the fractional distributions of isotopologues of glycolytic metabolites, G6P, F6P, 1,3BPG, 3PG PEP and lactate; **G-H**, those of PPP metabolites R5P and S7P, and **I-K**, those of nucleotides ATP/UTP and sugar nucleotides UDP-GlcNAc, respectively. Total fractions of ^{13}C scrambled (Scr) species are also given for G6P, F6P, lactate, and S7P, which were calculated by summing the fraction of individual Scr species (**1-5** for G6P and F6P; **1-2** for lactate; and **1-6** for S7P). In **A-H**, the fully ^{13}C labeled isotopologues were lower in fractions in the PDTX than in the corresponding ex vivo tissue models, which were opposite in trend to the unlabeled (**0**) and ^{13}C scrambled isotopologues. For R5P and S7P, the differences for the fully ^{13}C labeled isotopologues corresponded to the lower extent of enrichment in the $^{13}C_5$-isotoplogues (i.e. the ribose subunit) of ATP and UTP in PDTX versus ex vivo tissue models. All abbreviations were as in **Supplementary Figs. 2, 3 and 4**. The x-axis represents the number of ^{13}C atoms present in each isotopologue. Values shown were mean ± SEM (n=3). *, **, and *** denote p values for PDTX versus slice metabolites; <0.05-0.01, <0.01-0.001, and <0.001, respectively.

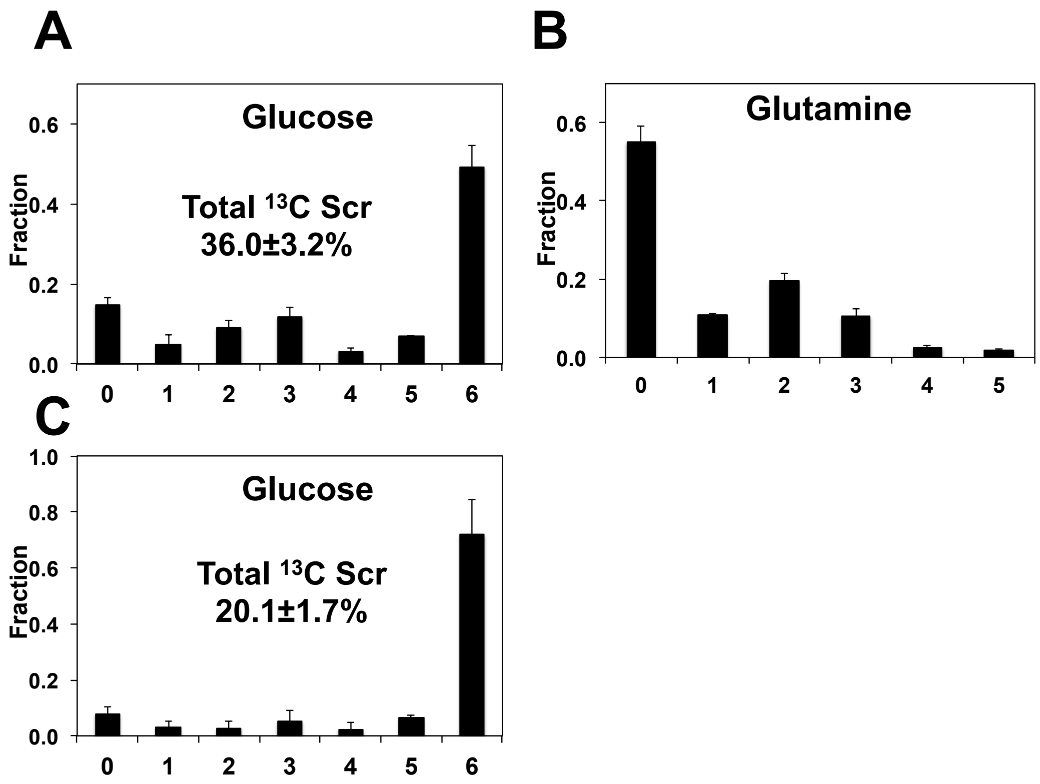

Supplementary Figure 9: UHR-FTMS analyses indicate the release of ^{13}C labeled Gln and ^{13}C scrambled glucose into the plasma of PDTX models.

Isotopologue distributions of plasma glucose (**A**) and glutamine (**B**) in PDTX mice were analyzed by nanoelectrospray UHR-FTMS, respectively without (glucose) and with (glutamine) ECF derivatization. Significant ^{13}C scrambling of glucose (total 36%) was observed, the pattern of which was similar to that of G6P in liver (**Supplementary Fig. 2A**). Isotopologue distribution of plasma glucose (**C**) in the BAsT cell xenografts mice were analyzed by nanoelectrospray UHR-FTMS. Significant ^{13}C scrambling of glucose (total 20%) was also observed in these mice. The x-axis represents the number of ^{13}C atoms present in each isotopologue represented in the text as $^{13}C_i$. Values shown are mean ± SEM (n=3).

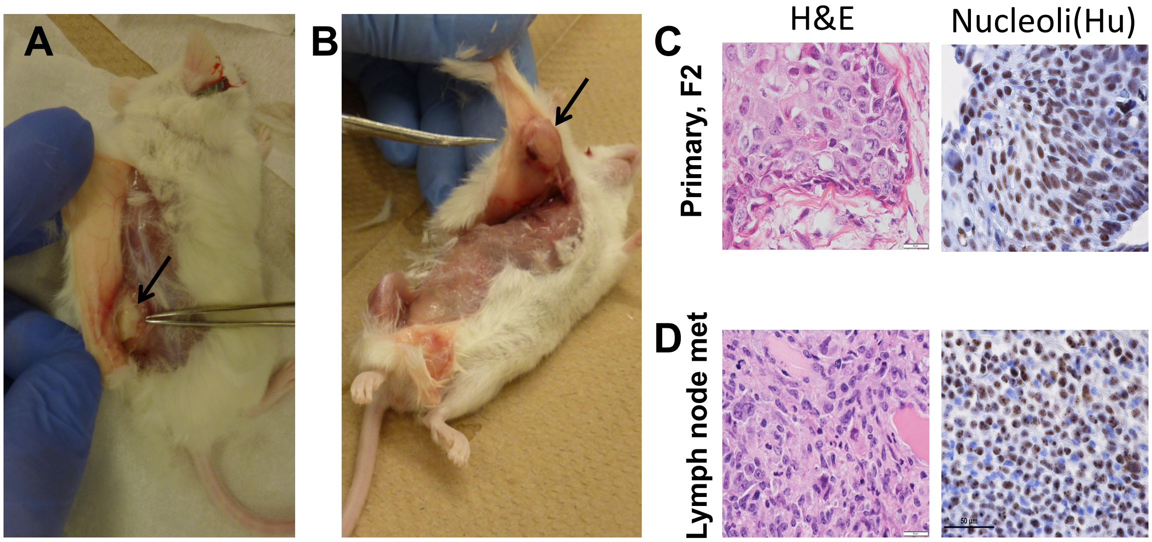

Supplementary Figure 10: Lymph node metastases are observed in a mouse PDX model of primary NSCLC.

Pieces of resected tumor (0.2 mm^3) were implanted each in the left and right shoulders of NSG mice within 30 min after surgery and was allowed to grow to 1 cm^3. The tumor-bearing mice (F0 generation) were fed $^{13}C_6$-glucose enriched liquid diet for 18 h before necropsy. A portion of the tumors were re-implanted into additional sets of mice for propagation to F2 generation, while the remaining tumor tissues were processed, extracted, and analyzed for ^{13}C labeling patterns of metabolites by IC-UHR-FTMS. We noticed the formation of large lymph node lesions (B, arrow) in all F2 mice carrying this PDTX (A, arrow). Liquid diet feeding of $^{13}C_6$-glucose, tumor harvest, and metabolite analysis were as described above for the F0 mouse tumors. **A**, **B**. gross morphology of F2 primary tumor and its lymph node metastatic lesion, respectively. **C**, **D**. H&E stain and immunohistochemical staining for human nucleoli (ab190710, Abcam) of the two lesions (400x magnification) showing the presence of human cells in the lesion.

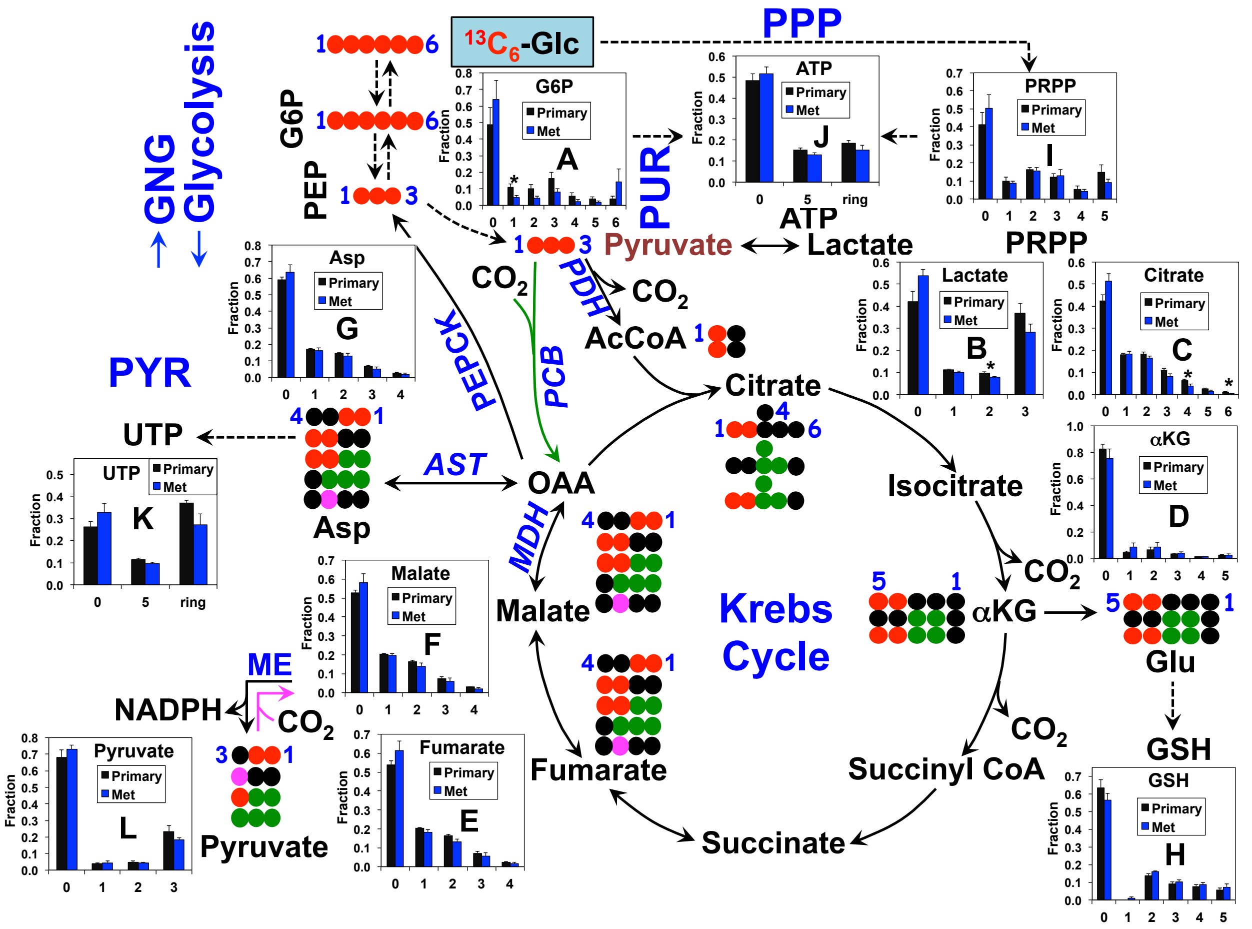

Supplementary Figure 11: Lymph node metastases show little changes in the fractional distribution of the isotopologue products of PPP and biosynthetic pathways of glutathione and nucleotides relative to the matched primary PDTX in the same mouse. Fractional enrichment of various isotopologues of representative central metabolites in the primary tumor and metastatic lymph node lesions was calculated from the data in **Fig. 4**. None of these isotopologues showed significant difference between the two tumor types of the same mouse. For GSH, PRPP, ATP, and UTP, this lack of changes in the fractional enrichment of the ^{13}C isotopologues from primary tumor to lymph node lesions was a result of a comparable increase in the pool sizes of both unlabeled and ^{13}C labeled isotopologues in the latter tumor type (cf. **Fig. 4**). Such pattern of changes in fractions and pool sizes suggest enhanced capacity for the synthesis of glutathione and nucleotides from both glucose and non-glucose sources or it could reflect decreased utilization of glutathione and nucleotides in metastatic versus primary tumors.

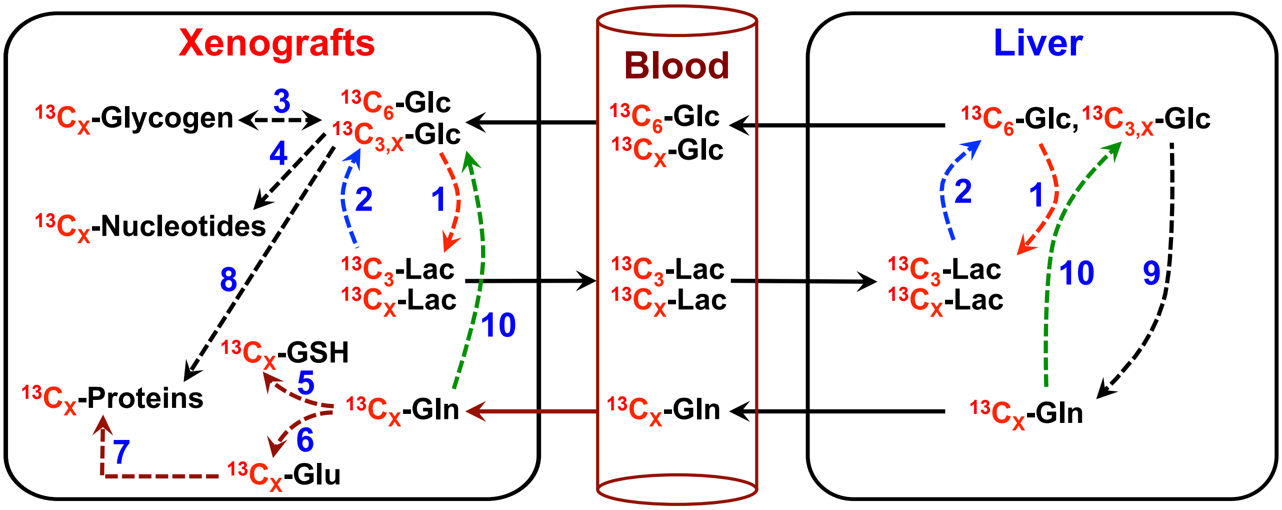

Supplementary Figure 12: Interactions of glucose metabolic networks in NSCLC PDTX and liver in vivo

The diagram illustrates glucose metabolic networks in PDTX and liver as well as their interactions via blood. **1**: glycolysis leading to $^{13}C_3$-lactate (Lac) production; **2**: gluconeogenesis from $^{13}C_3$-lactate or ^{13}C scrambled ($^{13}C_X$) lactate leading to the production of $^{13}C_3$- or other ^{13}C scrambled glucose); **3**: ^{13}C labeled glycogen synthesis from ^{13}C labeled glucose; **4**: de novo synthesis of purine and pyrimidine nucleotides from ^{13}C labeled glucose via glycolysis, pentose phosphate pathway, the Krebs cycle, one-carbon metabolism, and nucleobase synthesis; **5-7**: synthesis of ^{13}C-enriched glutathione (GSH), Glu, and proteins from ^{13}C-labeled Gln originated from blood; **8**: ^{13}C-enriched protein synthesis from ^{13}C-enriched glucose; **9**: ^{13}C- enriched Gln synthesis from ^{13}C-enriched glucose via glycolysis, the Krebs cycle, and Gln synthetase; **10**: synthesis of ^{13}C-scrambled glucose from ^{13}C- enriched Gln via glutaminolysis, the Krebs cycle, and gluconeogenesis.

Supplementary References

1 Moseley, H. N. Correcting for the effects of natural abundance in stable isotope resolved metabolomics experiments involving ultra-high resolution mass spectrometry. *BMC Bioinformatics* **11**, 139, doi:10.1186/1471-2105-11-139 (2010).

2 Lane, A. N., Fan, T. W. M., Xie, Z., Moseley, H. N. B. & Higashi, R. M. Isotopomer analysis of lipid biosynthesis by high resolution mass spectrometry and NMR. *Analytica chimica acta* **651**, 201-208, doi:10.1016/j.aca.2009.08.032 (2009).

3 Yang, Y., Fan, W. W.-M., Lane, A. N. & Higashi, R. M. Chloroformate Derivatization for Tracing the Fate of Amino Acids in Cells by Multiple Stable Isotope Resolved Metabolomics (mSIRM). *Anal. Chim. Acta* **976**, 63-73, doi:10.1016/j.aca.2017.04.014 (2017).
